# Death Receptor 3 (TNFRSF25) Increases Mineral Apposition by Osteoblasts and Region Specific New Bone Formation in the Axial Skeleton of Male DBA/1 Mice

**DOI:** 10.1155/2015/901679

**Published:** 2015-05-03

**Authors:** Fraser L. Collins, Jessica O. Williams, Anja C. Bloom, Michael D. Stone, Ernest Choy, Eddie C. Y. Wang, Anwen S. Williams

**Affiliations:** ^1^Institute of Infection and Immunity, School of Medicine, Cardiff University, Cardiff CF14 4XN, UK; ^2^University Hospital Llandough, Cardiff & Vale University Health Board, Cardiff CF64 2XX, UK

## Abstract

*Objectives*. Genome wide association studies identified TNFSF member TNF-like protein 1A (TL1A, TNFSF15) as a potential modulator of ankylosing spondylitis (AS). TL1A is the only confirmed TNFSF ligand of death receptor 3 (DR3, TNFRSF25); however, its role in disease pathology is not characterised. We evaluated DR3's role in controlling osteoblast- (OB-) dependent bone formation* in vitro* and* in vivo*.* Methods*. Osteoprogenitor cells and OB were cultured from male DR3-deficient (DR3^ko^) and wild-type (DR3^wt^) DBA/1 mice. DR3 and RANKL expression were tested by flow cytometry. Alkaline phosphatase and mineralization were quantified. Osteopontin, osteoprotegerin, and pro MMP-9 were measured by ELISA. A fluorescent probe (BoneTag) was used to measure* in vivo* mineralization in 10-month-old mice.* Results*. DR3 was expressed on osteoprogenitors and OB from DR3^wt^ mice. Alkaline phosphatase, osteopontin, and mineral apposition were significantly elevated in DR3^wt^ cultures. Levels of RANKL were comparable whilst osteoprotegerin was significantly increased in DR3^wt^ cultures.* In vivo* incorporation of BoneTag was significantly lower in the thoracic vertebrae of 10-month-old DR3^ko^ mice.* Conclusions*. These data identify new roles for DR3 in regulating OB-dependent bone mineral apposition. They potentially begin to explain the atypical pattern of new bone formation observed in the axial skeleton of grouped, aging DBA/1 mice.

## 1. Introduction

In a normal physiological state the process of bone remodeling is accomplished through the coupled activities of the osteoclast (OC) and the osteoblast (OB). The OC is responsible for the degradation of bone while the OB produces the organic matrix and aids in its mineralization [1, 2]. In certain pathological conditions, however, OC and OB activity can become uncoupled leading to an increase or decrease in bone resorption and bone formation. Increased bone formation is observed in the spondyloarthritides; delicate marginal syndesmophytes are common in ankylosing spondylitis (AS) whilst nonmarginal syndesmophytes are frequently observed in reactive arthritis and psoriatic arthritis (PsA) [3–5]. The cause of this excessive focal bone formation by OB is currently not clear. The immune system and increased expression of proinflammatory cytokines such as members of the tumour necrosis factor super family (TNFSF) and their receptors have been shown to be implicated [3]. Our study assesses the impact of the TNF receptor superfamily (TNFRSF) member death receptor 3 (DR3) upon OB-directed mineral apposition with a view to understanding how it modulates bone remodelling in the axial skeleton.

Identification of a link between the DR3/TL1A pathway and the spondyloarthritides was first demonstrated when single-nucleotide polymorphisms (SNPs) located in the direct vicinity of the* TNFSF15* gene were shown to be strongly associated with predisposition to spondyloarthropathy [6]. Further evidence for a role of the DR3/TL1A pathway in AS was provided by Konsta et al., who identified that serum levels of TL1A are significantly elevated in AS patients over healthy controls [7]. The mechanism through which the DR3/TL1A pathway potentially drives the pathogenesis of the spondyloarthritides, however, is not clear. Expanded numbers of circulating CD8^+^ CD28^−^ T cells have been identified in AS patients, which correlated with a more severe course of disease [8]. A study by Twohig et al., demonstrated that TL1A signalling on TCR stimulated CD8^+^ T cells resulted in increased proliferation [9]. The indirect effect of DR3/TL1A signalling in CD8^+^ T cells upon the expansion of OBs or mineral apposition by OB, however, has not been resolved. Studies by Bu et al. [10] and Borysenko et al. [11] demonstrated the expression of DR3 on the surface of a human OB cell line and primary human OB. These findings imply that the DR3/TL1A signaling pathway could directly modulate apposition of bone matrix by OB, a notion that has not been investigated previously.

This study is the first to specifically investigate the functional role of DR3 on mineral apposition by OB* in vitro* and* in vivo* using male DBA/1 mice lacking the DR3 gene (DR3^ko^). The spontaneous development of ankylosing enthesopathy has been reported previously in aging male DBA/1 mice and our study provides the first insight to DR3's role in controlling new bone formation by OB in the axial skeleton [12]. Here we report that OB-derived DR3 increases expression of the early OB differentiation marker alkaline phosphatase, increases expression of the transcription factors Runx2 and OSX, and regulates mineral apposition. MMP-9 and OPN production by OB were measured as surrogate markers of increased OB activation. OB and OC cross talk is an important factor in bone homeostasis. We measured OB-derived RANKL and OPG production to determine the role of DR3 in regulating OC differentiation by OB. These findings examine potentially important, hitherto unknown, mechanisms whereby DR3 regulates OB-dependent bone homeostasis.

## 2. Materials and Methods

### 2.1. Animals

All experiments were undertaken in male DBA/1 DR3^wt^ and DR3^ko^ mice generated in a DR3^het^ × DR3^het^ colony. The DBA/1 DR3^ko^ strain was generated through backcrossing C57BL/6 DR3^het^ mice [13] with DBA/1 wild-type mice for 7 generations. All DBA/1 DR3^wt^ and DR3^ko^ animals were generated in-house. Animals used for* in vitro* experiments were 8–12 weeks of age. Animals for the* in vivo* BoneTag experiments were 10 months of age. Animals were housed in filter top cages at a constant temperature and humidity on a 12-hour light/dark cycle. Food and water were available* ad libitum*. All procedures were approved by the Local Research Ethics Committee and performed in strict accordance with Home Office approved license PPL 30/2361.

### 2.2. OPC and OB Cell Culture

To obtain osteoprogenitors (OPCs) bone marrow was isolated from the femora of DR3^wt^ (*n* = 5) and DR3^ko^ (*n* = 5) DBA/1 mice and cultured in *α*MEM supplemented with 20% foetal calf serum, 50 *μ*g/mL Penicillin-Streptomycin (control medium) and 5% CO_2_ at 37°C. At confluence OPCs were detached from the culture vessel by scraping and were replated in a 12-well plate at a density of 4 × 10^4^ cells/well in control medium. Osteoblasts were subsequently differentiated from OPCs by addition of *α*MEM supplemented with 10% foetal calf serum, 50 *μ*g/mL Penicillin-Streptomycin, 10 mM *β*-glycerophosphate, 50 *μ*g/mL ascorbic acid, and 10 nM dexamethasone (mineralisation medium). Osteoblasts were maintained for up to 26 days (5% CO_2_ at 37°C) with medium changes every 3-4 days.

### 2.3. Cellular Staining and Flow Cytometry

OPCs (DR3^wt^ *n* = 6, DR3^ko^ *n* = 4) and OB (DR3^wt^ *n* = 3, DR3^ko^ *n* = 2) were cultured for 15 days using the method described above. Cells were removed from the culture vessel and incubated with Fc block (BD Pharmingen, CA, USA) for 15 min followed by a 30 min block with 2% normal goat serum. Cells were stained with either polyclonal *α*-mouse DR3-biotin (BAF 2437; R&D Systems, MN, USA) followed by SA-APC (Molecular Probes, OR, USA) or PE-conjugated *α*-mouse RANKL mAb (IK22/5; Santa Cruz Biotechnology Inc., TX, USA). Data were acquired on a BD Accuri C6 flow cytometer and analysed with CFlow software (BD Biosciences).

### 2.4. TL1A RT-PCR

RNA was extracted from OPCs and mineralising OB (DR3^wt^ *n* = 1, DR3^ko^ *n* = 1) using RNeasy (QIAGEN) following manufacturer's instructions and converted to cDNA using SuperScript II Reverse Transcriptase (Life Technologies). RT-PCR was performed according to standard Life Technologies protocols. PCR primers were as follows: TL1A, forward 5′-CAG CAG AAG GAT GGC AGA-3′ and reverse 5′-CTC TGG CCT GTG TCT ACA-3′, giving a 91-bp product; and *β*-actin, forward 5′-CGG CCA GGT CAT CAC TAT TG-3′ and reverse 5′-CTC AGT AAC CCG CCT AG-3′, giving a 450-bp product. The PCR comprised 33 cycles with an annealing temperature of 59°C. The PCR products were size fractionated by agarose gel electrophoresis and analysed using a UVP BioDoc It imaging system (Cambridge, UK).

### 2.5. Quantitative PCR

RNA was extracted from OPCs and OB and converted to cDNA as described above (DR3^wt^ *n* = 1, DR3^ko^ *n* = 1). Quantitative PCR was performed using the iCycler iQ system (Bio-Rad) with RT2 Real Time SYBR green PCR master mix (SA Biosciences) following the recommended protocol for SYBR green (Bio-Rad). Assays were run in duplicate. mRNA levels were determined using the comparative C_t_ method and normalized to *β*-actin mRNA levels. Primers used for quantitative PCR were as follows: RUNX2, forward 5′-CCCTGAACTCTGCACCAAGT-3′ and reverse 5′-TGGCTCAGATAGGAGGGGTA-3′; OSX, forward 5′-TCTCCATCTGCCTGACTCCT-3′ and reverse 5′-CAGGGGACTGGAGCCATAGT-3′; *β*-actin as above.

### 2.6. Alkaline Phosphatase and Mineralisation Staining

Alkaline phosphatase (ALP) activity was determined in OB cultures (DR3^wt^ *n* = 5, DR3^ko^ *n* = 5) using SigmaFast BCIP/NBT stain (Sigma). The mineralized matrix was stained for calcium deposition by Alizarin-red staining. Briefly, at time points indicated in results, cultures were fixed with a 4% formaldehyde/PBS solution for 15 min. Cultures were then incubated with either SigmaFast BCIP/NBT solution or Alizarin-red (1% solution in water) for 30 minutes. Cultures stained for mineral apposition were washed with 50% methanol to remove nonspecific background. Plates were air-dried and scanned and the percentage of the well was covered by ALP positive cells or mineral calculated with Image J (NIH).

### 2.7. Measurement of Soluble Mediators in Cell Culture Supernatant

Expression of soluble mediators in cell culture supernatants was analysed by ELISA using mouse osteoprotegerin (OPG), pro MMP-9, receptor activator of nuclear factor kappa-B ligand (RANKL) and osteopontin (OPN) DuoSet kits (all R&D Systems).

### 2.8. *In Vivo* Imaging

DBA/1 male mice were housed together as previously described [12]. Animals at 10 months (DR3^wt^ *n* = 3, DR3^ko^ *n* = 3) of age were subjected to either an intraperitoneal injection with IRDye 680 BoneTag optical probe (0.08 nmol/g) or equal volume of PBS as control. After 24 hours animals were sacrificed by CO_2_ asphyxiation. Images were taken using the Xenogen IVIS 200 (excitation wavelength 676 nm, emission wavelength 698 nm, f-stop 1) and rectangular regions of interest of fixed dimensions were selected at the thoracic spine, knee, and tail. Total efficiency of the marker was analyzed using the Live Image software, version 2.50.2 (Caliper). The efficiency values obtained from the PBS control animals were subtracted from the values obtained with BoneTag, to account for background autofluorescence.

### 2.9. Statistical Analysis

Data are expressed as mean ± SEM and statistical analysis performed using the Mann-Whitney *U* test for nonparametric data, unpaired *t*-test for parametric data, or 2-way analysis of variance (ANOVA) using GraphPad Prism software (GraphPad, San Diego, CA, USA). *P* values of ≤0.05 were considered significant and values of ≤0.01 were considered highly significant.

## 3. Results

### 3.1. Expression of DR3 and TL1A by OPCs and OB

**Figure 1:**
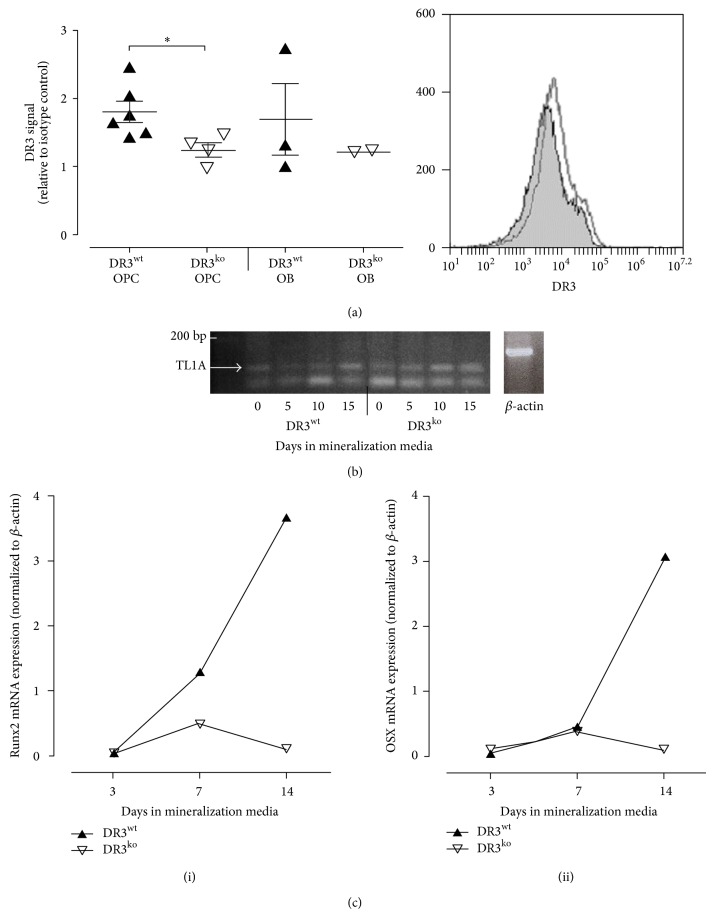
Expression of DR3 by murine OPCs and OBs. (a) DR3^wt^ (*n* = 6) and DR3^ko^ (*n* = 4) OPCs and OB (DR3^wt^ *n* = 3, DR3^ko^ *n* = 2) were analysed for DR3 signal by flow cytometry. DR3 signal was observed on DR3^wt^ OPCs and OBs. A small subpopulation of DR3^wt^ OPCs displayed higher levels of DR3 signal (shaded area = isotype control, black outline = DR3 antibody). (b) RNA was extracted from DR3^wt^ and DR3^ko^ OPCs and OB and tested for* TL1A* mRNA by RT-PCR. A 91 bp band corresponding to TL1A mRNA was detected at all time-points demonstrating constitutive expression of* TL1A* mRNA by DR3^wt^ and DR3^ko^ OPCs and OB. (c) Samples (DR3^wt^ *n* = 1, DR3^ko^ *n* = 1) were tested by qPCR at time points indicated to determine whether DR3 signalling modulates (i) Runx2 or (ii) OSX gene expression. Increased expression of Runx2 was detected at day 7 and day 14 while increased OSX expression was only detected at day 14 in the DR3^wt^ compared to the DR3^ko^.

Expression of DR3 on the human MG63 osteosarcoma cell line has previously been demonstrated by Borysenko et al. [11]. Here we investigated DR3 expression on primary, murine OPCs and OB using flow cytometry. The signal from the surface of DR3^wt^ OPCs was 1.8 ± 0.2-fold higher than the isotype control (Figure 1(a)) and was significantly higher than from DR3^ko^ OPCs (*P* ≤ 0.05). Levels of DR3 signal were reduced on DR3^wt^ mineralising OB (1.4 ± 0.8-fold increase) compared to the OPCs. RT-PCR analysis of DR3^wt^- and DR3^ko^-derived OPCs and OB showed constitutive expression of* TL1A* mRNA across a 15 day time course (Figure 1(b)).

### 3.2. DR3/TL1A Signalling Promotes* RUNX2* and* OSX* Expression

To identify whether DR3 signalling in OPCs and OB modulates Runx2 and OSX expression qPCR was performed on differentiating OB across a 14-day time course (Figure 1(c)). No difference in* RUNX2* and* OSX* levels was detected between the DR3^wt^ and DR3^ko^ cultures after the first 3 days of differentiation. At day 7 an increase in Runx2 gene expression was observed in the DR3^wt^ cultures. At day 14 both Runx2 and OSX expression levels were elevated in the DR3^wt^ cultures compared to the DR3^ko^ cultures.

### 3.3. Effect of DR3 on OB Differentiation, OPN and Pro MMP-9 Expression and Mineral Apposition

**Figure 2:**
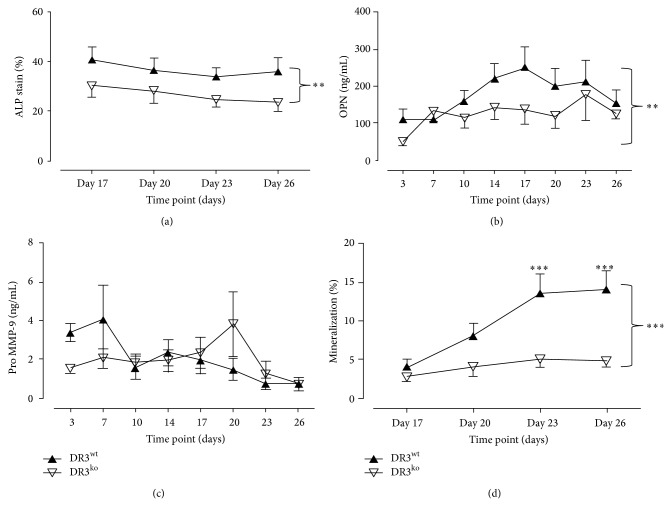
Expression of ALP, OPN, pro MMP-9, and mineral apposition in DR3^wt^ and DR3^ko^ osteoblast cultures. (a) DR3^wt^ (*n* = 5) and DR3^ko^ (*n* = 5) derived OB were cultured and the percentage of well area covered by ALP^+^ cells was calculated. ALP staining was significantly higher in the DR3^wt^ compared to the DR3^ko^ cultures across the 26-day time course (*P* ≤ 0.01). Supernatants from OB cultures were tested for soluble levels of (b) OPN and (c) pro MMP-9. Across the time course significantly increased levels of OPN were detected in DR3^wt^ versus DR3^ko^ cultures (*P* ≤ 0.01). (d) Levels of mineral apposition in the cultures were tested by alizarin-red staining. Significantly increased levels of mineralization were observed in the DR3^wt^ cultures at days 23 and 26 (*P* ≤ 0.001) compared to the DR3^ko^. This translated into a significant increase in the level of mineral apposition across the 26-day time course (*P* ≤ 0.001).

To assess the effect of DR3 on OB differentiation and mineral apposition, mineralization assays were performed and expression of the early OB differentiation marker ALP was studied. At the earliest time point assessed (day 17), the percentage area of the wells demonstrating ALP-positive cells was elevated in the DR3^wt^ (41 ± 5%) versus DR3^ko^ (30 ± 5%) cultures. These levels did not vary significantly across the 26-day time course, translating into significantly increased levels of ALP staining in DR3^wt^ cultures (*P* ≤ 0.05; Figure 2(a)).

To see whether the increase in DR3^wt^ ALP expression was accompanied by an increase in OB activation markers, levels of the Ca^2+^ binding osteoid protein (OPN) and a regulator of bone matrix maturation (pro MMP-9) were measured in culture supernatants [14, 15]. Levels of OPN were significantly elevated in DR3^wt^ cultures compared to DR3^ko^ cultures over the 26-day time course (*P* < 0.01; Figure 2(b)). Levels of OPN peaked at day 17 in the DR3^wt^ (252 ± 55 ng/mL) and at day 23 in the DR3^ko^ cultures (178 ± 70 ng/mL). Levels of pro MMP-9 were initially higher at day 3 in the DR3^wt^ cultures versus DR3^ko^ (3.4 ± 0.5 ng/mL versus 1.5 ± 0.2 ng/mL; *P* < 0.01). DR3^wt^ pro MMP-9 levels peaked at day 7 (4.1 ± 1.7 ng/mL) and decreased over the duration of the time course to 0.7 ± 0.3 ng/mL. In contrast, expression of pro MMP-9 in the DR3^ko^ cultures increased over the culture period and peaked at day 20 (3.8 ± 1.7 ng/mL; Figure 2(c)).

To identify whether the increase in DR3^wt^ OB differentiation and OPN expression correlated with an effect on mineral apposition, levels of mineralization in the cultures were evaluated. At day 17 comparable levels of mineralization were observed between the DR3^wt^ (4 ± 1%) and DR3^ko^ (3 ± 1%) cultures. Mineral apposition was significantly increased in the DR3^wt^ cultures at day 23 (14 ± 2%; *P* < 0.001) and day 26 (14 ± 2%; *P* < 0.001) when compared to DR3^ko^ cultures (5 ± 1% and 5 ± 1%, resp.). This resulted in significantly increased levels of mineral apposition across the time course in the DR3^wt^ cultures (*P* < 0.001; Figure 2(d)). Having demonstrated that DR3 affects OB differentiation and mineral apposition,* in vitro*, we investigated whether it could have a potential effect on OB control of osteoclast formation.

### 3.4. Effect of DR3 on OB-Derived RANKL and OPG

**Figure 3:**
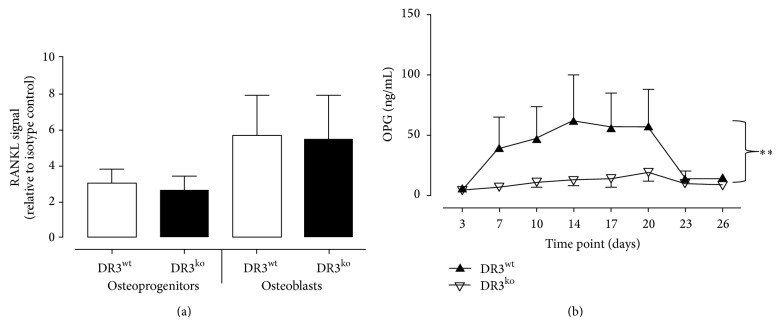
Effect of DR3 on OB-derived RANKL and OPG. (a) Cell surface expression of RANKL was analyzed on DR3^wt^- and DR3^ko^-derived OPCs and OB by flow cytometry. Both DR3^wt^ and DR3^ko^ OPCs displayed a 3-fold increase in RANKL signal over isotype control. RANKL signal increased to 5-fold over isotype in DR3^wt^ and DR3^ko^ OB. (b) Levels of soluble OPG were measured in culture supernatants by ELISA (DR3^wt^ *n* = 5, DR3^ko^ *n* = 5). Expression of OPG was significantly elevated in the DR3^wt^ compared to DR3^ko^ OB cultures over the 26-day time course (*P* ≤ 0.01).

To investigate whether DR3 had a role in regulating OB control of OC formation, levels of OB-derived RANKL and OPG were studied. OPC and OB cell surface RANKL expression were comparable between DR3^wt^ and DR3^ko^ cells (Figure 3(a)). No soluble RANKL was detected in any culture supernatants (data not shown). In contrast, significantly increased levels of OPG were detected across the 26-day time course in DR3^wt^ compared to DR3^ko^ cultures (*P* < 0.01; Figure 3(b)). Levels of OPG peaked at day 14 (62 ± 37 ng/mL) in DR3^wt^ cultures, while in DR3^ko^ cultures OPG levels reached a peak of 19 ± 7 ng/mL on day 17.

### 3.5. Assessing the Impact of DR3 on Bone Formation* In Vivo*

**Figure 4:**
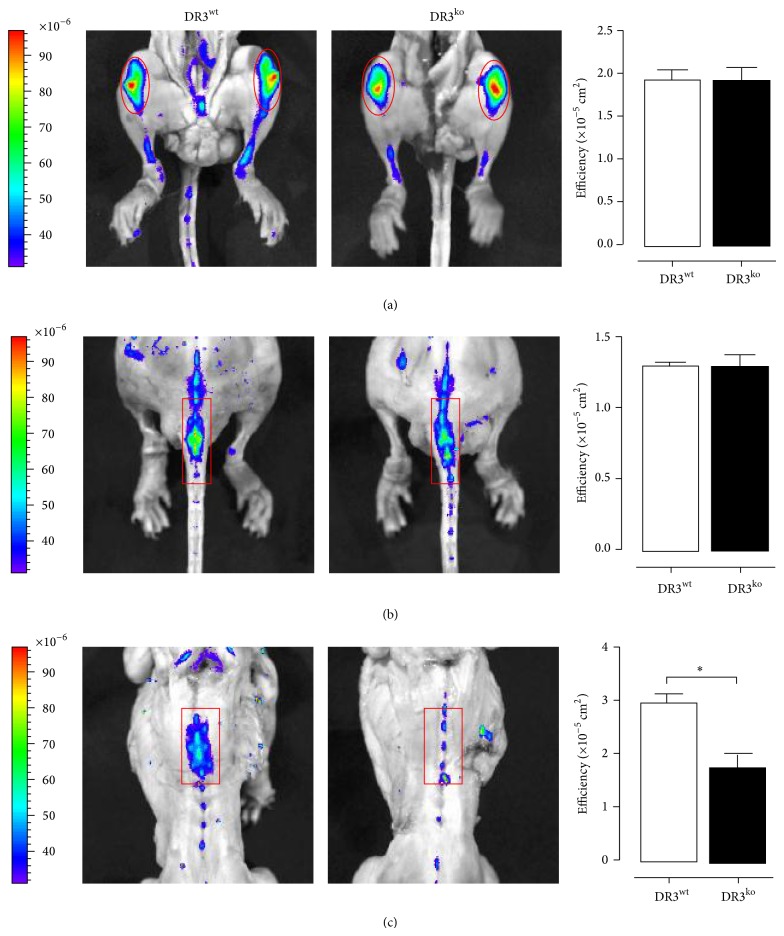
Incorporation of BoneTag into the knee, tail, and thoracic spine of 10-month-old DR3^wt^ and DR3^ko^ mice (DR3^wt^ *n* = 3, DR3^ko^ *n* = 3). Mice were injected with the fluorescent probe BoneTag and* in vivo* bone formation was analyzed. No differences in bone formation were observed in the (a) knee or (b) tail. A significant increase in bone formation was observed in the (c) thoracic spine of DR3^wt^ compared to DR3^ko^ mice (*P* ≤ 0.05). Area quantified illustrated by red circle/box.

*In vitro* DR3 has been shown to regulate OB differentiation and mineral apposition while promoting expression of the antiosteoclastogenic cytokine OPG. To identify what effect this would have on* in vivo* bone formation male DBA/1 mice were aged for 10 months as a spontaneous model of ankylosing enthesopathy [12]. Bone formation over a 24-hour period was measured at three sites (knee, tail, and thoracic vertebra) via the incorporation of the fluorescent probe BoneTag. Significantly increased fluorescence efficiency values were observed in the DR3^wt^ thoracic spine (2.94 ± 0.20 × 10^−5^; *P* < 0.05) compared to the DR3^ko^ thoracic spine (1.76 ± 0.26 × 10^−5^; Figure 4(c)), suggesting DR3^ko^ mice exhibit some resistance to the long-term development of spontaneous ankylosing enthesopathy observed in DBA/1 strain wild-type animals.

## 4. Discussion

Spondyloarthritides such as AS and PsA are characterized by the formation of syndesmophytes [3–5]. Mechanisms that control this increased aberrant bone apposition are not clear. This study provides the first direct evidence of DR3's involvement in OB-dependent new bone formation* in vitro* and* in vivo*. We demonstrate (using DR3^ko^ mice) that DR3 has an important role in the differentiation of OB and subsequent mineralization of the organic matrix by OB. We also reveal a novel pathway whereby DR3 increases OPG production by OB thereby potentially decreasing OC differentiation.* In vivo*, we show that gene deletion of DR3 protects the axial skeleton against excessive bone formation, a bone phenotype previously observed in aging DBA/1 male mice [12].

In the current study we reveal that DR3 is expressed on the cell surface of primary murine OPCs and OBs. DR3 expression on mouse OPC was shown to be comparable to that reported on the human OB MG63 cell line [11]. Cell surface expression of DR3 was reduced on mineralizing OB suggesting that DR3 may be temporally regulated during OB differentiation. In addition to DR3, the current study identified for the first the time expression of TL1A mRNA by primary OPCs and OB. These data suggest a potential autocrine role for the DR3/TL1A pathway in modulating OB differentiation and mineral apposition.

The notion that DR3 signalling has a role in OB differentiation was supported by the mineralization assay results. Levels of ALP expression and mineral apposition were significantly increased in the DR3^wt^ cultures. This could be explained by DR3 signalling increasing either OB differentiation or proliferation, as has been described for effector T cells [16–18]. Total OB cell numbers at experiment end-point were, however, comparable between the DR3^wt^ and DR3^ko^ cultures (data not shown), suggesting that the increase in ALP expressing cells was due to DR3 promoting differentiation. This result is at odds with data described by Borysenko et al. [11], who demonstrated that cross-linking of DR3 with an antibody on MG63 cells induced apoptosis and inhibited differentiation. However, Borysenko and colleagues were unable to reproduce their results with TL1A or in primary human OB, suggesting that activation following receptor cross-linking may differ from actions of the natural ligand. Further support for DR3 having a role in the regulation of OB differentiation is provided by the analysis of Runx2 and OSX gene expression. Runx2 and OSX are critical transcription factors involved in OB differentiation, the absence of which results in the maturational arrest of the OB [19–21]. In the present study both Runx2 and OSX were elevated in the DR3^wt^, but not DR3^ko^, cultures at later time points. These data suggest a possible mechanism through which OB-dependent DR3 signalling potentially modulates OB differentiation.

The DR3-dependent increase in OB differentiation in the DR3^wt^ was associated with increasing mineral apposition in the cultures. This elevation in mineral apposition corresponded with a significant increase in OPN expression in the DR3^wt^ cultures. OPN is suggested to play a role in osteogenesis by attachment of the OB to the organic matrix and to regulate crystal size during mineralization [14, 22]. Interestingly OPN is in part transcriptionally regulated by Runx2 [22, 23] suggesting that in OBs, DR3 modulates OPN expression indirectly via this transcription factor. Before the OB can commence with the formation of newly mineralized bone the organic matrix must first undergo a maturation process. In a study by Filanti et al. [15] members of the matrix metalloproteinase (MMP) family, including MMP-2, -9, and -14, were increased in OB cultures at the time of nodule formation suggesting that they play a significant role in this matrix maturation process. Previous studies have demonstrated that DR3 signalling has a role in MMP-9 production; the THP-1 cell line has been shown to increase MMP-9 expression following treatment with TL1A [24, 25]. In addition, a study by Wang et al. [26] described significantly lower levels of MMP-9 in the joints of DR3^ko^ mice with antigen induced arthritis compared to their DR3^wt^ counterparts during the early stages of disease. In the present study we sought to determine whether OB-dependent DR3 signalling affected osteoblastic MMP-9 expression, which would potentially modulate OB mineral apposition. A difference in the MMP-9 expression pattern was observed between the DR3^wt^ and DR3^ko^ cultures; with higher levels of pro MMP-9 observed at the early time points in the DR3^wt^ cultures. This early expression of MMP-9 shows similarity to the phenotype described by Wang et al. [26] and taken together the results suggest that DR3 signalling has a central role in controlling levels of MMP-9. Unpublished work from our laboratory also suggests that the DR3 pathway may have a key role in the activation of MMP-9 from the pro form.

In addition to an OB specific effect, we have shown* in vitro* that OB DR3-dependent signalling results in significantly elevated expression of the soluble RANKL inhibitor OPG. This data suggests that OB DR3 signalling has the potential to in-directly modulate OC formation; where the increased OB-derived OPG inhibits OC formation and subsequently bone resorption [27]. This is in contrast to the direct contribution of DR3 signalling on osteoclastogenesis during inflammatory arthritis where DR3 has been revealed to enhance OC formation [28]. Interestingly, significantly increased levels of OPG have been detected in the serum from AS patients and are linked to poor physical mobility [29].

To investigate whether DR3 regulation of OB differentiation and mineral apposition could contribute towards the increased bone formation associated with the spondyloarthritides, we examined the effect of DR3 on* in vivo* bone formation in the spontaneous ankylosing enthesopathy model [12]. The results from the 10-month-old mice are consistent with the hypothesis that OB-dependent DR3 signalling has a significant role in regulating the atypical bone formation observed in the axial skeleton of mice with spontaneous ankylosing enthesopathy. The increased bone formation observed in the thoracic spine of DBA/1 DR3^wt^ mice is analogous to the pattern of marginal/nonmarginal syndesmophyte formation observed in the spine of PsA and AS patients [30, 31]; thus this observation could have important implications in the understanding of the pathogenesis of these diseases.

The results from the present study provide the first evidence for a functional role for DR3 signalling in new bone formation. We have shown that cell surface DR3 is temporally expressed by OPCs and OB while TL1A mRNA is constitutively expressed and that an autocrine signalling loop may promote Runx2 and OSX expression, consequently modulating OB differentiation and mineral apposition. OB-dependent DR3 signalling increased levels of the soluble inhibitor OPG and could, therefore, indirectly regulate OC formation and bone resorption. Finally,* in vivo*, the absence of DR3 leads to differences in bone formation compared to wild-type DBA/1 mice, which in 10-month-old animals suggest resistance to the development of spontaneous ankylosing enthesopathy that has been previously described in this strain [12]. These data, taken with the study by Konsta et al. [7], suggest that OB-dependent DR3 signalling could be an important contributory factor in the atypical bone pathology associated with diseases such as PsA and AS.
